# Cdk5 Regulates Accurate Maturation of Newborn Granule Cells in the Adult Hippocampus

**DOI:** 10.1371/journal.pbio.0060272

**Published:** 2008-11-11

**Authors:** Sebastian Jessberger, Stefan Aigner, Gregory D Clemenson, Nicolas Toni, D. Chichung Lie, Özlem Karalay, Rupert Overall, Gerd Kempermann, Fred H Gage

**Affiliations:** 1 Laboratory of Genetics, The Salk Institute for Biological Studies, La Jolla, California, United States of America; 2 Institute of Cell Biology, Department of Biology, ETH Zurich, Zurich, Switzerland; 3 Helmholtz Zentrum München, German Research Center for Environmental Health, Munich, Germany; 4 Center for Regenerative Therapies (CRTD), Dresden, Germany; Cincinnati Children's Hospital, United States of America

## Abstract

Newborn granule cells become functionally integrated into the synaptic circuitry of the adult dentate gyrus after a morphological and electrophysiological maturation process. The molecular mechanisms by which immature neurons and the neurites extending from them find their appropriate position and target area remain largely unknown. Here we show that single-cell–specific knockdown of cyclin-dependent kinase 5 (cdk5) activity in newborn cells using a retrovirus-based strategy leads to aberrant growth of dendritic processes, which is associated with an altered migration pattern of newborn cells. Even though spine formation and maturation are reduced in cdk5-deficient cells, aberrant dendrites form ectopic synapses onto hilar neurons. These observations identify cdk5 to be critically involved in the maturation and dendrite extension of newborn neurons in the course of adult neurogenesis. The data presented here also suggest a mechanistic dissociation between accurate dendritic targeting and subsequent synapse formation.

## Author Summary

Neural stem cells divide and generate new neurons throughout life in the mammalian hippocampus. After a distinct maturation process, newborn neurons become functionally integrated into the preexisting circuitry and appear to participate in hippocampal function, which is critically involved in certain forms of learning and memory. However, the molecular mechanisms by which new neurons find their position and project to their appropriate target area remain largely unknown. We here show that cell-type–specific reduction of cyclin-dependent kinase 5 (cdk5) activity in newborn neurons results in impaired neuronal migration and leads to the extension of incorrectly targeted neuronal processes. Strikingly, ectopic processes extending from newborn cells synaptically integrate, suggesting a dissociation between accurate targeting of processes extending from newborn neurons and subsequent synapse formation, which might have important implications for the restorative use of neural stem cells in neurological disease.

## Abstract 3

New neurons are born throughout life in the hippocampus. Cell-type-specific, single-gene ablation in newborn neurons reveals that the kinase cdk5 regulates growth of neuronal processes, identifying a key mechanism of neuronal integration within the adult brain.

## Introduction

New granule cells are added into the dentate circuitry throughout life, as neurogenic neural stem and progenitor cells (NPCs) persist in the adult hippocampus [1]. Hippocampal NPCs give rise to only one neuronal subtype, excitatory dentate granule cells, in contrast to the other neurogenic area of the adult brain, the subventricular zone (SVZ)/olfactory bulb, where several subtypes of inhibitory neurons are born [2]. Dentate granule cells are highly polarized, glutamatergic neurons that receive their main excitatory input onto dendrites extending into the molecular layer (ML) and that send out axons targeting pyramidal cells in area CA3, as well as inhibitory basket cells, interneurons, and excitatory mossy cells [3–5]. Despite growing knowledge regarding the cellular and molecular mechanisms of fate choice instruction of NPCs in vivo [6–9] and the description of several developmental steps during the integration, selection, and maturation process of adult-generated neurons [10–12], the regulatory genes required for neuronal maturation and neurite pathfinding of newborn granule cells remain largely unknown.

It seems plausible that there is a certain degree of conservation between the molecular pathways used during embryonic and early postnatal development, and the integration of new granule cells in the adult brain [9,13,14]. However, an obvious difference between embryonic and early postnatal development in one case and the integration of newborn granule cells into the preexisting dentate circuitry in the other is that adult neurogenesis is a heterogeneous and dynamic process, with cells in all stages of maturation at any given time [15]. For example, previously suggested models of axonal growth that predict a concerted repulsion by chemorepellents expressed at a certain time postnatally are difficult to apply to the mechanisms of pathfinding and integration of new neurons in the mature hippocampus [16–18]. Given that alterations in hippocampal neurogenesis might be key components in hippocampus-associated neurological diseases, such as major depression [19], Alzheimer disease [20], and epilepsy [21], understanding the molecular mechanisms underlying neuronal migration, neurite extension, and pathfinding of newborn neurons would seem important to gain further insights into neurological disease.

By analyzing quantitative trait loci derived from recombinant inbred mice [22], we have previously identified several gene loci that may harbor genes critically involved in the process of adult neurogenesis. One of the candidate loci was a region on mouse chromosome 5 containing the cyclin-dependent kinase 5 (cdk5) gene. Cdk5 is a highly versatile kinase that requires association with its regulatory partner, p35, for activation [23], and plays a pivotal role in a variety of neurobiological processes, such as neuronal migration, neurite extension, dendritic pathfinding, homeostatic synaptic plasticity, neuronal degeneration, dopamine signaling, and learning and memory [24–34].

Here, we used a retrovirus-based approach for a cell-type–specific knockdown of cdk5 activity in newly generated granule cells born in the adult hippocampus. We show that cdk5 is critically involved in migration, dendritic pathfinding, and neuronal maturation of newborn granule cells. Thus, our findings identify a molecular pathway that governs the accurate spatial integration of newborn neurons in the adult dentate gyrus.

## Results

### Cdk5 Activity Is Not Required for Proliferation or Differentiation of NPCs In Vitro

**Figure 1:**
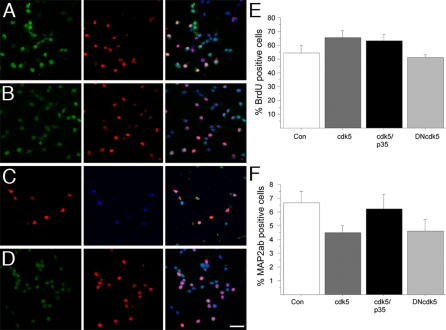
Cdk5 Gain- or Loss-of-Function Does Not Affect the Proliferation and Differentiation Capacity of Adult NPCs (A–E) Stable transduced cell lines expressing a control virus (A), *cdk5* (B), *cdk5* plus coelectroporation with *p35* (red in [C]), and *DNcdk5* (D) showed no statistically significant differences in BrdU (red in [A, B, and D], blue in [C]) uptake after a 1-h pulse (E). Percentages of BrdU-labeled cells (E) were 54.5 ± 5.3% (Con), 65.7 ± 4.9% (cdk5), 63.2 ± 4.7% (cdk5/p35), and 51.0 ± 2.2% (DNcdk5, *p* > 0.2). Left and middle panels in (A–D) show single channels for GFP (green), BrdU (red in [A, B, and D]; blue in [C]), and p35/CAG-RFP coelectroporation (red in [C]). Merged images in (A, B, and D) also include DAPI (blue). Note that the total number of GFP-labeled cells expressing *p35* decreased due to electroporation-associated toxicity, whereas the relative proportion of BrdU-labeled cells was not altered with *p35* electroporation. (F) Stable transduced cell lines expressing a control virus, *cdk5*, or *DNcdk5* were treated for 4 d with retinoic acid and forskolin to induce neuronal differentiation. There were no significant differences upon neuronal induction between the groups (*p* > 0.08). Percentages of MAP2ab-expressing cells were 5.8 ± 0.8% (Con), 4.5 ± 0.5% (cdk5), 6.3 ± 1.0% (cdk5/p35), and 4.6 ± 0.8% (DNcdk5). Scale bar in (D) represents 50 μm. Error bars represent the standard error of the mean (s.e.m.).

We first analyzed the expression profile of cdk5 in the course of neuronal differentiation of NPCs isolated from rat hippocampus and mouse whole brain. Consistent with a specific role for cdk5 in neuronal cells, we found that, in proliferating rat and mouse NPCs, cdk5 mRNA and protein were expressed at low levels but were robustly up-regulated upon induction of neuronal differentiation (Figure S1A and unpublished data). To analyze the functional involvement of cdk5 in NPC proliferation and/or neuronal differentiation, we generated stably transduced lines of adult mouse- and rat-derived NPCs overexpressing the wild-type cdk5 protein or a well-characterized kinase-deficient cdk5 mutant that acts as a dominant-negative (*DNcdk5*, [23]). Overexpression of *cdk5* (either alone or with its coactivator, *p35*; [23]) or inhibition with *DNcdk5* had no significant effects (*p* > 0.2) on the proliferation of adult rat NPCs (Figure 1A–1E) or mouse NPCs (unpublished data), as measured by the number of bromo-deoxy-uridine (BrdU)-incorporating cells after a 1-h pulse.

**Figure 2:**
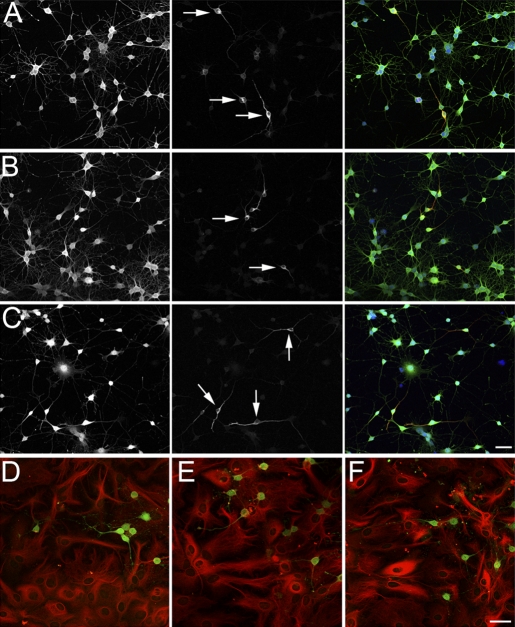
Cdk5 Gain- or Loss-of-Function Does Not Affect Differentiation Capacity of Adult NPCs (A–C) Stable transduced cell lines expressing a control virus (A), *cdk5* (B), or *DNcdk5* (C) were treated for 4 d with retinoic acid and forskolin to induce neuronal differentiation. Shown are single-channel images for GFP (left panels), MAP2ab (middle panels), and merged images (including DAPI, right panels). Arrows point toward MAP2ab-expressing neuronal cells. There were no significant differences between the groups upon neuronal induction (see Figure 1F). (D–F) Cocultures of NPCs with hippocampal astrocytes induced neuronal differentiation, but no substantial morphological differences were found after 12 d of coculture between control (D), *cdk5*-expressing (E), and *DNcdk5*-expressing (F) cells. The scale bar in (C), which applies to (A–C), and the scale bar in (F), which applies to (D–F), represent 50 μm.

Likewise, gain- or loss-of-function of cdk5 activity did not substantially alter the neuronal differentiation capacity in rat (Figures 1F and 2A–2C) or mouse NPCs (unpublished data), because the number of MAP2ab-labeled neuronal cells was not significantly different between control cells and cells expressing *cdk5* or *DNcdk5* (*p* > 0.08) after induction of neuronal differentiation. Since we had previously shown that hippocampal astrocytes instruct NPCs to adopt a neuronal fate [8], we sought to confirm these findings in cocultures of rat NPCs with hippocampal astrocytes. Cells overexpressing *cdk5* or *DNcdk5* were indistinguishable from control cells, and they acquired a neuronal morphology (Figure 2D–2F). Thus, we conclude that cdk5 function is not critical for in vitro NPC proliferation or differentiation.

### Cdk5 Deficiency Impairs Dendritic Targeting In Vivo

**Figure 3:**
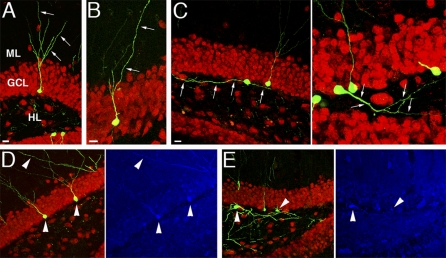
Cdk5 Deficiency Leads to Aberrant Dendritic Processes in Newborn Granule Cells (A–C) Four weeks after intrahippocampal injection of a GFP-expressing retrovirus (GFP, green) to label adult-generated granule cells (NeuN, red in [A–C]) control cells (A) had a highly polarized morphology with a single apical dendrite extending towards and branching within the ML (arrows in [A]). *Cdk5* overexpression had no substantial effects on granule cell morphology 4 wk after injection (B). Retrovirus-mediated inhibition of cdk5 activity with a dominant-negative kinase mutant (*DNcdk5*) altered dendritic morphology and resulted in dendrites traveling along the GCL (left panel in [C]) or extending into the hilus (right panel in [C]). Arrows in (A–C) point to dendritic processes. (D and E) Cdk5 and DNcdk5 expression levels (blue in right panels in [D and E]) were selectively increased in retrovirus-transduced, GFP-labeled neurons expressing NeuN (red in left panels in [D and E], arrowheads mark examples of virus-labeled cells). HL, hilus. Scale bars in [A–C] represent 10 μm.

Based on these observations, we reasoned that cdk5 might function in advanced stages of neuronal maturation and integration, which might be revealed only when NPCs are embedded in their physiological niche. We therefore examined the role of cdk5 in newborn neurons within the adult dentate gyrus. *Cdk5* mRNA is expressed throughout the hippocampus, with strong expression in the adult dentate area (Figure S1B). We overexpressed *cdk5* or *DNcdk5* in newborn cells using a retroviral strategy that delivers a transgene, along with green fluorescent protein (GFP) as a label, to dividing NPCs and their progeny [5,35]. Four weeks after transduction with control virus expressing GFP alone, newborn cells consistently showed a highly polarized morphology, with a single apical dendrite branching in the outer parts of the dentate granule cell layer (GCL) and extending through the ML (Figure 3A). Overexpression of *cdk5* or its coactivator *p35* (unpublished data) did not substantially change neuronal morphology 4 wk after virus injection (Figure 3B and 3D). Importantly, newborn control cells or *cdk5*-overexpressing cells never extended dendritic processes towards the hilus. In striking contrast, 51.3 ± 9.8% of newborn neurons over-expressing *DNcdk5*, and thus inhibiting cdk5 kinase activity, lost the polarity typical of dentate granule cells, and extended dendrites along the GCL or even towards the hilus when analyzed 4 wk after viral transduction (Figure 3C and 3E). Confirming overexpression of retrovirus-expressed genes, we visualized both cdk5 and DNcdk5 (recognized by the cdk5 antibody) using immunohistochemistry. Cdk5 (Figure 3D) and DNcdk5 (Figure 3E) showed high expression levels in cell somata and dendritic processes extending from newborn cells 4 wk after viral injection, highlighting the robust and cell-type–specific expression of the transgenes in newborn cells. To confirm the effects of decreasing cdk5, we phenocopied the effect of functional cdk5 deficiency on the dendritic morphology of newborn granule cells by small interfering RNA (siRNA)-mediated reduction in *cdk5* levels, using a retrovirally expressed small hairpin RNA (shRNA) directed to the *cdk5* message (Figure S2).

**Figure 4:**
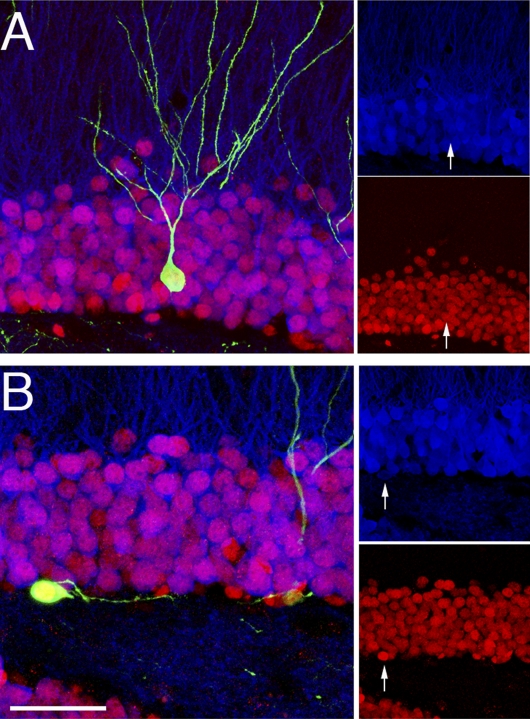
Aberrant Cdk5-Deficient Neurons Express Granule Cell Markers Control cells (A) and *DNcdk5*-overexpressing cells (B) colabeled with the granule cell markers Prox-1 (red) and Calbindin (blue), demonstrating that newborn neurons were dentate granule cells. Small panels in (A and B) show the single channels for Prox-1 (red) and calbindin (blue). Arrows point towards the GFP-labeled, newborn cells. HL, hilus. Scale bar in (B) represents 50 μm.

**Figure 5:**
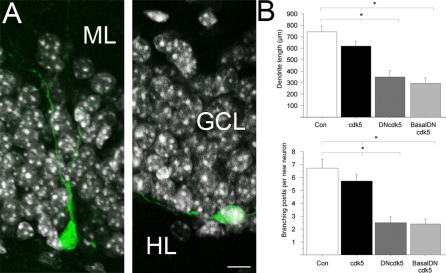
Cdk5 Deficiency Affects Early Steps of Neuronal Maturation and Alters Dendritic Structure (A) One-week after retroviral transduction, control cells extended dendritic processes towards the ML (left panel). In contrast, *DNcdk5*-overexpressing cells (right panel) extended processes traveling along the GCL that were not oriented towards the ML. Scale bar represents 10 μm. (B) *DNcdk5* expression altered dendritic orientation and led to reduced dendritic length and decreased branching points, whereas *cdk5* overexpression had no effect on dendritic complexity. Dendritic lengths were 740.7 ± 53.2 μm (Con), 619.7 ± 43.2 μm (cdk5), 347.2 ± 60.3 μm (DNcdk5), and 297.3 ± 59.3 μm (BasalDNcdk5). Error bars represent s.e.m. A single asterisk (*) indicates *p* < 0.05. HL, hilus.

Despite their dramatic morphological abnormality, newborn neurons overexpressing *DNcdk5* expressed the granule cell-specific marker, Prox-1, and the Ca^2+^-binding protein of mature granule cells, calbindin, as did CAG-GFP–labeled control cells (Figure 4), indicating that no change in neuronal fate had occurred. Morphological changes induced by *DNcdk5* overexpression occurred early during the neurogenic process: as early as 7 d after viral transduction, the orientation of dendritic processes was altered (Figure 5A).

### Cdk5 Deficiency Alters Dendritic Morphology

Given the morphological alterations of newborn granule cells in vivo, we next analyzed whether cdk5 might regulate not only dendritic targeting, but also the complexity of dendrites. We traced control, *cdk5*-overexpressing, and *DNcdk5*-overexpressing cells and measured the total dendritic length and number of branching points 4 wk after virus injections. Since the penetrance of the DNcdk5 phenotype (i.e., extending aberrantly targeted dendrites) was approximately 50%, we grouped *DNcdk5*-expressing newborn granule cells into cells with ML-targeted dendrites (DNcdk5) and cells whose dendrites failed to extend into the ML (BasalDNcdk5). Cdk5 inhibition with *DNcdk5* reduced the number of dendritic branching points and total dendritic length 4 wk after retroviral injection irrespective of the position of dendrites within the dentate area, whereas retroviral overexpression of *cdk5* had no effect on dendritic architecture (Figure 5B and 5C). Dendritic length of newborn cells expressing *DNcdk5* was already reduced 2 wk after virus injection, suggesting that impaired growth, rather than enhanced dendritic pruning, is the underlying cause for dendrite length reduction (Con 232 ± 63.1 μm, DNcdk5 117.9 ± 39.3 μm, and BasalDNcdk5 108.0 ± 46.4 μm; *p* < 0.05). These data show that cdk5 is critical for the development of proper dendritic architecture.

### Cdk5 Is Involved in Spine Formation of Newborn Granule Cells

**Figure 6:**
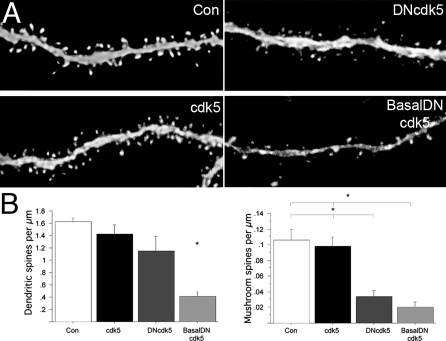
Inhibition of Cdk5 Activity Attenuates Dendritic Spine Formation and Maturation (A) Shown are confocal images of spines extending from control cells (Con), *cdk5*-expressing cells (cdk5), *DNcdk5*-expressing granule cells with correctly targeted dendrites in the ML (DNcdk5), and aberrantly targeted dendrites toward the hilus (BasalDNcdk5). (B) Inhibition of cdk5 activity did not affect the formation but decreased the maturation of dendritic spines on apical dendrites of *DNcdk5*-expressing granule cells, whereas *DNcdk5*-expressing neurons with aberrantly targeted basal dendrites (BasalDNcdk5) showed a decrease in both formation and maturation of dendritic spines. In contrast, retroviral overexpression of *cdk5* did not affect spine formation or maturation compared to control cells. Error bars represent s.e.m. A single asterisk (*) indicates *p* < 0.05.

Following up on previous reports analyzing the function of cdk5 in dendritic maturation and spine formation in vitro or during embryonic and early postnatal development [36–38], we next asked whether cdk5 was not only involved in dendritic targeting and complexity, but might also be critical for spine formation and maturation. Therefore, we analyzed the density and shape of spines from control cells and *cdk5*-overexpressing or *DNcdk5*-overexpressing cells. Again, *DNcdk5*-expressing newborn granule cells were grouped into cells with ML-targeted dendrites (DNcdk5) and cells whose dendrites failed to extend into the ML (BasalDNcdk5). We found no difference in spine density or number of mushroom spines between control and *cdk5*-overexpressing cells (Figure 6A and 6B). The number of spines extending from correctly targeted *DNcdk5*-expressing cells was also not different from control numbers. However, the density of spines on dendrites extending from *DNcdk5*-overexpressing cells that failed to extend dendrites into the ML (BasalDNcdk5) was reduced compared to control cells (Figure 6). Interestingly, both correctly and aberrantly targeted dendrites from *DNcdk5*-expressing cells showed reduced numbers of mushroom spines compared to newborn cells labeled with a control virus.

### Aberrant Dendrites from Cdk5-Deficient Neurons Receive Synaptic Input

**Figure 7:**
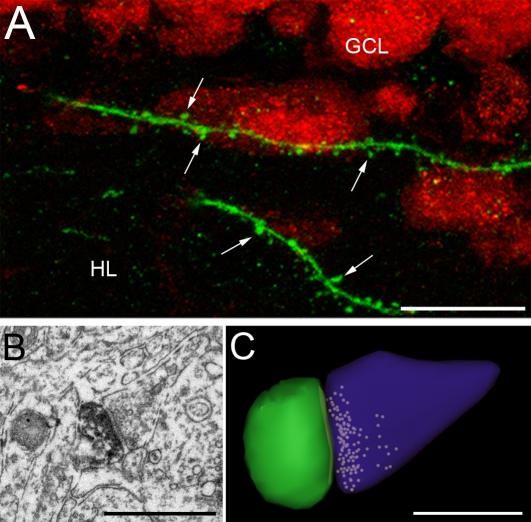
Synaptic Integration of Cdk5-Deficient Newborn Granule Cells Extending Aberrant Dendrites (A) Confocal microscopy revealed that aberrant dendrites extending from *DNcdk5*-expressing granule cells (arrows in [A]) traveling along the GCL (NeuN, red) were covered with spiny processes. (B and C) Serial section electron microscopy showed synapse formation as defined by the presence of the following on at least one section: a clear postsynaptic density, at least four presynaptic vesicles within 100 nm of the presynaptic membrane, and a well-defined synaptic cleft. Note the postsynaptic density of the GFP-labeled, electron-dense dendritic spine and opposing vesicles in the axon terminal. A three-dimensional reconstruction of the synapse shown in (B) is depicted in (C). The scale bar in (A) represents 10 μm, and the scale bars in (B and C) represent 0.5 μm. HL, hilus.

Despite aberrant dendritic targeting, dendrites of *DNcdk5*-expressing newborn granule cells extending into the hilus were decorated with dendritic spines (Figures 6 and 7A). The existence of spines suggested that incorrectly targeted dendrites became synaptically integrated into the dentate circuitry despite their aberrant localization. In support of this notion, we consistently found close apposition of the presynaptic protein, synapsin, with spines of dendrites extending from *DNcdk5*-expressing cells that were located in the hilus, similar to dendritic spines in the ML extending from control cells and *cdk5*-overexpressing cells (Figure S3). To obtain independent evidence of synapse formation, we analyzed spines of *DNcdk5*-expressing newborn granule cells extending aberrant dendrites at the ultrastructural level [3]. We used serial section electron microscopy and identified spines from *DNcdk5*-expressing hilar dendrites that appeared to form bona fide synapses (Figure 7B and 7C).

### Altered Neuronal Migration Pattern in Cdk5-Deficient Newborn Granule Cells

**Figure 8:**
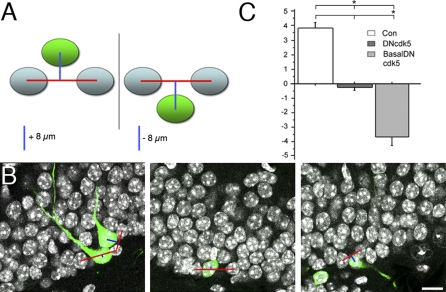
Cdk5 Deficiency Attenuates Migration of Newborn Neurons (A and B) The positions of newborn granule control cells 4 wk after birth and cells expressing *DNcdk5* were measured in relation to neighboring granule cells as depicted in (A) The centers of the nuclei of neighboring granule cells were connected and a perpendicular line was drawn toward the center of the GFP-labeled newborn cell. The distance from the nucleus of the GFP-labeled cell to the line connecting the center of the two neighboring nuclei was calculated with a positive (toward the ML) or negative (toward the HL) prefix. *DNcdk5*-expressing neurons were grouped into cells with correctly (DNcdk5) and aberrantly (BasalDNcdk5) targeted dendritic processes. (B) shows examples of confocal images that were used to measure distances from neighboring granule cells (left panel: Con, middle panel: DNcdk5, and right panel: BasalDNcdk5). Scale bar in (B) represents 10 μm. (C) Four-week-old newborn cells labeled with a control virus migrate on top of their neighboring cells. The migration was reduced (DNcdk5) or absent (BasalDNcdk5) in cdk5-deficient cells. HL, hilus. Error bars represent s.e.m. A single asterisk (*) indicates *p* < 0.05.

Cdk5 inhibition thus led to aberrant neurite extension and ectopic synapse formation. We next analyzed the migration pattern of newborn granule cells with attenuated cdk5 activity. In contrast to the SVZ of the lateral ventricles, from where newborn neurons migrate via the rostral migratory stream into the olfactory bulb, there is no clear evidence for a migrational route of newborn granule cells in the dentate gyrus. Previous studies have shown that the vast majority of adult-generated cells stay within the inner third of the GCL [39,40]. To analyze the position of newborn granule cells in more detail, we measured the position of newborn cells in relation to neighboring granule cells 4 wk after viral injection (Figure 8A and 8B). Newborn cells labeled with a control virus migrated on average 3.83 ± 0.66 μm deep into the GCL (Figure 8B and 8C). Strikingly, newborn granule cells expressing *DNcdk5* and extending aberrant dendrites were consistently located below the GCL (−3.60 ± 0.74 μm, *p* < 0.01). In contrast, the fraction of *DNcdk5*-expressing cells that had dendrites growing toward the ML showed some migration into the GCL (0.28 ± 0.36 μm), suggesting that impaired migration is associated with aberrant neurite extension (Figure 8B and 8C).

### Cdk5 Deficiency Only Mildly Affects Neuronal Survival In Vivo

**Figure 9:**
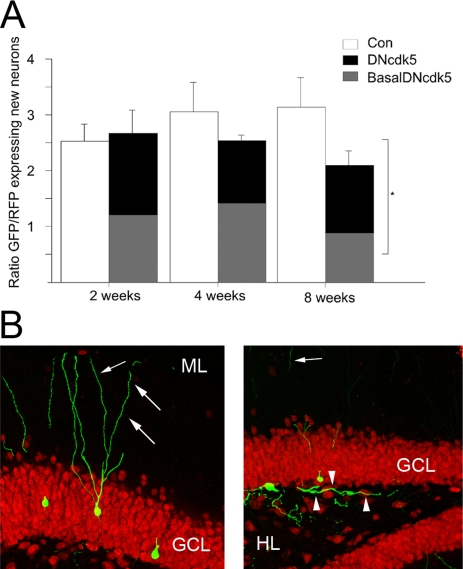
Cdk5 Deficiency Moderately Affects the Survival of Newborn Neurons (A) The ratio of control and *DNcdk5*-expressing cells with correctly targeted dendrites (DNcdk5) was not changed 2, 4, and 8 wk postinjection. No significant reduction was found for *DNcdk5*-expressing newborn granule with aberrant dendrites (BasalDNcdk5) 2 and 4 wk after injection compared to control cells. A small but significant decrease in the number of surviving cells appeared 8 wk postinjection. Error bars represent s.e.m. A single asterisk (*) indicates *p* < 0.05. (B) One year after the injection of a control virus, GFP-labeled, newborn granule cells (green, left panel) expressing NeuN (red) were stably integrated into the dentate circuitry and extended dendrites into the ML (arrows). Despite abnormal dendritic morphology (arrowheads), GFP-labeled *DNcdk5*-expressing granule cells (green, right panel) could be identified even 1 y after virus injection. HL, hilus.

It has been shown previously that the survival of newborn granule cells in the adult dentate gyrus is an activity-dependent selection process [10]. Given this finding, we next asked if the survival of newborn granule cells deficient in cdk5-kinase activity is altered. We analyzed the number of retrovirally labeled newborn granule cells at several time points after intrahippocampal coinjections of either the *DNcdk5*-overexpressing retrovirus or the GFP-expressing control virus, together with another control retrovirus encoding the red fluorescent protein (RFP). This strategy allowed us to analyze the dynamics in the number of newborn cells, largely irrespective of variations in virus titer and injection sites. We found that, 2 and 4 wk postinjection, the number of cdk5 activity-deficient neurons was not affected (*p* > 0.2 and *p* > 0.09, respectively), and only a small decrease in surviving *DNcdk5*-expressing granule cells with aberrant dendrites appeared 8 wk after viral transduction (*p* < 0.05, Figure 9A). However, even 1 y after virus injection, aberrant *DNcdk5*-overexpressing granule cells still existed in the dentate area (Figure 9B), suggesting that, despite a moderately increased elimination rate of *DNcdk5*-overexpressing granule cells, a substantial portion of *DNcdk5*-expressing cells survived for extended periods of time. To determine whether the positioning or aberrant growth of dendrites extending from *DNcdk5*-expressing cells predicted neuronal survival, we again measured the relative position into the GCL of newborn cells compared to neighboring cells. Newborn cells labeled with a control virus were significantly deeper in the GCL at 8 wk compared to their position 4 wk postinjection (5.76 ± 0.96 μm, *p* < 0.05). Newborn granule cells expressing *DNcdk5* and extending aberrant dendrites remained below the GCL (−2.91 ± 0.59 μm).

## Discussion

We here used a cell-type–specific, retrovirus-based strategy to characterize the function of cdk5 in the context of adult hippocampal neurogenesis. A substantial fraction of cdk5-deficient newborn granule cells failed to extend dendritic processes towards the appropriate target zone of the ML and instead formed aberrant synaptic connections in the hilar area. The findings presented here identify a critical role for cdk5 in the migration and morphological maturation of newborn granule cells within the adult dentate gyrus and suggest a mechanistic dissociation between dendritic targeting and subsequent synaptic integration.

Previous work has established that newborn granule cells become synaptically and functionally integrated into the dentate circuitry [5,41,42]. Approximately 7-d-old newborn neurons start to extend dendritic processes toward the ML, where their dendrites ramify [4]. Four weeks after birth, newborn neurons show the highly polarized morphology typical of granule cells [4]. GABA-mediated depolarization, reelin signaling, Disc1 activity, and neurotrophic factors, such as brain-derived neurotrophic factor (BDNF) and their downstream signals, have been implicated in neurite extension and migrational behavior of newborn granule cells [13,43–46]. However, the signals that regulate the correct positional extension of newly formed neurites are unknown. We here show that a substantial fraction of newborn granule cells deficient in cdk5 activity (using dominant-negative and shRNA-mediated knockdown of cdk5) fail to send dendrites toward the ML, but extend neurites into the hilar region or along the GCL, suggesting a failure of the dendrites of newborn cells to find their appropriate target region in the ML. This finding is consistent with earlier observations that cdk5 is required for proper dendritic development of pyramidal neurons during embryonic cortical development [34]. Furthermore, genetic deletion of *p35*, which is a neuron-specific regulatory subunit of cdk5 required for cdk5 activity [23], leads to the formation of aberrant dendrites extending from granule cells born during embryonic and early postnatal development [47–49]. These previously described phenotypes of cdk5/p35 ablation support our results indicating that cdk5 is critically involved in proper dendrite extension from granule cells born in the adult dentate gyrus.

Interestingly, status epilepticus (SE), which is the most commonly used rodent model for temporal lobe epilepsy, induces, among other effects on neurogenesis, the formation of basal dendrites that become integrated into the dentate circuitry, which is not observed under physiological conditions [50–52]. The abnormal extension of dendrites after SE indicates a vulnerable phase for the initiation of aberrant growth of neurites extending from granule cells born in the adult hippocampus. Nevertheless, it remains unknown whether the effects of SE on newborn neurons are cell autonomous or due rather to seizure-associated changes in the dentate area niche. Our results presented here show that aberrant dendrites can develop with inhibition of cdk5 activity in a normal wild-type niche because only newborn cells are genetically modified by retroviral vectors. These results also point to a cell-autonomous effect of cdk5 on neuronal maturation.

Notably, not only the position, but also the length, of dendrites extending from cdk5-deficient newborn granule cells was altered. In principle, there are two possibilities to explain this observation: (1) reduced dendritic growth is secondary to the abnormal position of dendrites, or (2) attenuation of cdk5 activity itself impairs dendritic growth directly. Our finding that dendrite length was reduced not only in hilar dendrites, but also in dendritic processes that extended correctly from *DNcdk5*-expressing granule cells supports previous reports that cdk5 is involved in BDNF-stimulated dendritic growth [37], a mechanism that might also regulate dendrite extension in the context of adult neurogenesis [53,54]. Dendritic length of cdk5-deficient cells was not only reduced at 4 wk, but also at 2 wk after virus injection, suggesting impaired growth and not altered dendritic pruning as the underlying cause for reduced dendritic complexity. In addition, future studies will have to address whether altered presynaptic input in response to cdk5-deficiency might contribute to the dendritic phenotype observed in *DNcdk5*-expressing cells.

Strikingly, aberrant dendrites extending from *DNcdk5*-expressing granule cells were covered with dendritic spines, which are the common sites for glutamatergic synapses within the adult brain. Our results showing spine extension and synapse formation using light and electron microscopy indicate synaptic integration of aberrant dendrites into the preexisting circuitry. Formation of the first dendritic spines occurs approximately 16 d after newborn granule cells are born [4]. Interestingly, the tips of filopodia, thin processes that presumably become stabilized and transform into spines, are found in close vicinity to preexisting axonal boutons that already synapsed on other granule cells more frequently than random chance would predict [3,55]. Furthermore, axodendritic synapses of newborn granule cells seem to synapse preferentially onto preexisting boutons, suggesting a competitive mechanism of synapse formation and integration within the ML [3]. At this time, it remains unclear whether ectopic synapses formed on aberrant cdk5-deficient dendrites undergo similar steps as nascent synapses under normal conditions.

The data presented here provide evidence of a functional dissociation between dendritic targeting and subsequent synapse formation. The finding that hilar dendrites extending from *DNcdk5*-expressing granule cells form synapses suggests that dendritic filopodia/spines might be sufficient to induce axodendritic synapse formation within the adult brain. However, it is also possible that ectopic synapses mature in a similar way to newborn granule cells, with correctly targeted dendrites forming, at least transiently, multiple synapse boutons [3].

Interestingly, the number of spines was only reduced in aberrant dendrites, whereas spine density in dendrites extending from *DNcdk5*-expressing granule cells that targeted the ML were not reduced compared to controls. This finding suggests that cdk5 inhibition does not, per se, reduce the number of spines. However, cdk5 inhibition impaired maturation of dendritic spines into mushroom spines in aberrant as well as in correctly targeted dendrites, indicating an involvement of cdk5 in spine maturation. Notably, overexpression of cdk5 itself affected neither spine number nor shape of newborn granule cells. Together with previous reports that showed that hyperactivation of cdk5 by p25 enhances spine formation in CA1 pyramidal neurons [56], our findings show that cdk5 overexpression alone is not sufficient to increase the number of spines in newborn granule cells in the adult dentate gyrus but that cdk5 is required, irrespective of dendritic localization for spine maturation.

What might be the underlying cause of the aberrant extension of dendrites arising from *DNcdk5*-expressing granule cells? In accordance with previous reports [39,43], we found that newborn granule cells migrated short distances into the GCL under control conditions. Neuronal migration was impaired in *DNcdk5*-expressing granule cells that were extending aberrant dendrites compared to newborn *DNcdk5*-expressing cells with correctly targeted dendrites, indicating that impaired migration was associated with aberrant dendritic growth. There is no “hard measure” as to when migration of a newborn cell has “failed” (as there was also a small population of control cells that did not migrate into the GCL). This is clearly different from dendritic orientation, which is completely consistent in the group of newborn granule cells of the control groups: all of them extend a dendrite toward the ML. Therefore, the functional association between failed migration and aberrant dendrite extension remains unknown. Notably, cdk5 has a well-characterized function during neuronal migration. One interesting target protein of cdk5 in the context of adult neurogenesis is the microtubule-associated protein, doublecortin (DCX, [57,58]). Since DCX is transiently expressed in newborn granule cells [59,60] and we found that DCX and the cdk5-coactivator p35 coimmunoprecipitate in lysates of the adult hippocampus (unpublished data), the morphological changes caused by cdk5 inhibition might be associated with reduced phosphorylation of DCX. Alternatively, pathfinding of apical dendrites from newborn granule cells could be impaired by cdk5 inhibition. Indeed, previous reports have shown that cdk5 is involved in the semaphorin-3A–dependent extension of apical dendrites of glutamatergic principal neurons during cortical development [30,61]. Future studies will have to determine the downstream targets and upstream signals leading to altered migration and aberrant dendrite formation of *DNcdk5*-expressing, newborn granule cells. This search is complicated by the fact that the effects of cdk5 inhibition on migration and dendritic development in the context of adult hippocampal neurogenesis can best be adequately tested in vivo, within the context of the dentate area niche, as we found no substantial effects of cdk5 inhibition on cell proliferation and neuronal differentiation when we used NPCs in vitro.

The fact that aberrant dendrites are capable of forming synapses also has potential implications for transplantation-based approaches to treat neurological disease: our results indicate that aberrant neurites receive sufficient synaptic input leading to integration and long-term survival, which appears to require an activity-dependent mechanism in the adult hippocampus [10]. Thus, aberrant integration of transplanted cells that can be observed in subsets of transplanted neurogenic cells [62–65] might attenuate graft function or alter neuronal connectivity within the host brain. Interestingly, our experiments indicate that migration of newborn cells into the GCL is not a prerequisite for neuronal survival, as surviving *DNcdk5*-expressing cells consistently remained below the GCL even 8 wk after viral labeling.

The data presented here demonstrate a pivotal role for cdk5 in the neuronal maturation process of newborn granule cells, improving our understanding of molecular mechanisms that govern developmental steps from dividing NPCs to fully integrated newborn granule cells in the course of adult hippocampal neurogenesis.

## Materials and Methods

### Plasmids and retroviruses.

The in situ probe was isolated from a mouse whole-brain cDNA library using PCR primers against the 3′-UTR of cdk5 (corresponding to bp 1,066–1,667) and subcloned into pCRII vector (Invitrogen). CMV-driven expression constructs for *cdk5*, *DNcdk5*, and *p35*, and fusion construct cdk5-GFP were gifts from L. H. Tsai (Massachusetts Institute of Technology, Cambridge, Massachusetts). Expression constructs for *cdk5*, *DNcdk5*, and *p35* were subcloned into a retroviral vector driving the transgene under the chicken β-actin (CAG) promoter and containing an IRES-GFP (gift from I. Verma, Salk Institute, La Jolla, California). The shRNA against *cdk5* was cloned into pAmbion Silencer 2.0 for in vitro experiments. The target sequence was GCTGTACTCCACGTCCATC. For in vivo retroviral shRNA experiments, the shRNA was cloned into a vector containing an U6-promoter multiple cloning site and CAG promoter-driven GFP (gift from V. M. Sandler, Boston,Massachusetts). Control viruses were CAG-driven GFP and CAG-driven RFP viruses (RFP construct was a gift from R. Y. Tsien, University of California San Diego, La Jolla, California). Retroviruses were produced as described earlier [4]. Titers ranged between 1–5 × 10^7^ colony-forming units (cfu)/ml.

### Cell culture.

The rat and mouse NPCs used in this study have been described earlier [66]. For reverse transcriptase (RT)-PCR experiments, RNA was isolated using RNeasy (Qiagen) and cDNA was generated using the Superscript system (Invitrogen). Primer sequences for *cdk5*, *p35*, and *GAPDH* are available upon request. To produce stable transduced cell lines expressing control virus, *cdk5*-expressing, and *DNcdk5*-expressing virus, NPCs were transduced with the respective retrovirus and fluorescence activated cell sorting (FACS) was performed 4 d after transduction to yield pure virus-transduced cell populations. Electroporation of NPCs with the *p35* expression construct was performed as described earlier [6]. Electroporated cells were identified by coelectroporation with a CAG-RFP construct. Proliferation assays were performed with the addition of 1 μM bromo-deoxy-uridine (BrdU, Sigma-Aldrich) 1 h before fixation in FGF-2 (20 ng/ml). For differentiation studies, 1 μM retinoic acid (RA) and 5 μM forskolin (FSK) were added to the culture medium after the withdrawal of the mitogens. Cells were fixed 4 d later. The coculture experiments were performed as described before [8], plating approximately 2 × 10^3^ control, *cdk5*-expressing, or *DNcdk5*-expressing cells per square centimeter on a confluent astrocytic layer under serum-free conditions. Cells were fixed 12 d after plating. To quantify proliferation and differentiation, 500–1,000 cells were analyzed for labeling with BrdU or the neuronal marker MAP2ab. The investigator was blinded for the experimental conditions, and every experiment was performed with at least three biological replicates.

### Immunostaining, western blotting, and in situ hybridization.

Tissue and cells were fixed and processed for immunostaining as described earlier [41]. Primary antibodies used were rat α-BrdU (Harlan Seralab), mouse α-MAP2ab (Sigma), rabbit α-GFP (Molecular Probes), chicken α-GFP (Aves), rabbit α-Prox-1 (Chemicon), mouse α-Calbindin (Swant), rabbit α-synapsin (Calbiochem), mouse α-NeuN (Chemicon), rabbit α-cdk5 (Santa Cruz), rabbit α-p35 (Santa Cruz), and goat α-DCX (Santa Cruz). Secondary antibodies were obtained from Jackson Laboratory. Protein was isolated from 293T cells for siRNA testing 48 h after transfection of the respective constructs with Lipofectamine 2000 (Invitrogen), protein from rat NPCs was isolated under proliferating conditions and at the times indicated after addition of RA and FSK using RIPA buffer supplemented with protease inhibitors (Roche Complete EDTA-free supplemented with 0.2 mM 4-(2-aminoethyl) benzenesulfonyl fluoride hydrochloride). Proteins were separated by electrophoresis on 12% polyacrylamide gels, transferred to PVDF or nylon membranes, and probed overnight with rabbit α-Cdk5 (Santa Cruz Biotechnology) or rabbit α-p35 (Santa Cruz Biotechnology) antibody together with a mouse α-GAPDH (HyTest) antibody. α-Rabbit HRP-conjugated secondary antibodies (Pierce and Jackson) and α-mouse AP-conjugated (Promega) antibody were used at 1:5,000 dilution. Bands were detected by enhanced chemiluminescence. In situ hybridization was performed as described earlier [67] using 601-bp-long sense and antisense ^35^S-labeled riboprobes against cdk5. Sections were counterstained with bisbenzimide (Sigma). Sense probes detected no signal beyond unspecific background.

### Animals and retrovirus injections.

All animal procedures were performed in accordance with the protocols approved by the Animal Care Use Committee of the Salk Institute for Biological Studies. All mice in this study were 8- to 10-wk-old female C57Bl/6 mice. Mice were stereotactically injected with 1 μl of the *cdk5*- or *DNcdk5*-expressing viruses into the right dentate gyrus; the left side was always injected with a GFP-expressing control virus. Coordinates used were −2 a/p, ±1.5 m/l, from Bregma and −2.3 d/v from skull. For all experimental time points (1 wk, 4 wk, and 12 mo), the group sizes were *n* > 6. For survival studies, 0.75 μl of RFP-expressing control virus was coinjected with 0.75 μl of control GFP-, *cdk5*-expressing, or *DNcdk5*-expressing virus. Animals were killed 2, 4 and 8 wk later (*n* = 5 per group). We counted all virus-labeled cells using 1-in-6 series and formed a ratio between GFP- and RFP-expressing cells, allowing the estimation of the relative survival levels [10]. For shRNA experiments, six mice were injected with shRNA-expressing retrovirus into the right dentate gyrus, and control virus (with an empty U6-cassette) into the left dentate gyrus. Animals were killed 4 wk after viral injection.

### Migration analysis.

To analyze the position of newborn granule cells in relation to their neighboring cells, confocal images were acquired at the maximum extension of the soma of GFP-labeled cells. From these images, the center of the DAPI-labeled nucleus of the two adjacent cells to the GFP-labeled neuron were connected and a perpendicular line was drawn from the center of the DAPI-labeled nucleus of the GFP-expressing cells (see scheme in Figure 8A). Distances were measured using ImageJ. Analyses were performed 4 wk and 8 wk after viral injection. *DNcdk5*-expressing cells were grouped into cells that extended an apical dendrite toward the ML (DNcdk5) and cells that extended dendritic processes toward the hilus (BasalDNcdk5). For each condition, 15 randomly picked cells per mouse (*n* = 4) were analyzed.

### Dendritic structure and spine analysis.

Analyses of the dendritic structure were performed as described earlier [4]. Seven to 16 cells per experimental group were analyzed 2 and 4 wk after viral injection. The number and shape of spines were analyzed using at least six dendritic segments per animal from five mice per group 4 wk after retroviral injections, as previously described [4].

### Electron microscopy.

Mice were transcardially perfused with 4% paraformaldehyde in 0.1 M phosphate buffer (PB) (pH 7.4) at room temperature for 10 min. Fifteen hours after the perfusion was stopped, the brain was removed and postfixed for 48 h in 4% paraformaldehyde. Fifty-micrometer horizontal vibratome sections were then cut, cryoprotected in 2% glycerol and 20% DMSO in 0.1 M PB for 20 min, and freeze-thawed eight times in liquid nitrogen. After a treatment in 0.3% hydrogen peroxide (5 × 5 min) and three washes of 10 min in PB + 0.5% bovine serum albumin (BSA-C, Aurion), slices were incubated overnight in the primary antibody (rabbit α-GFP, Chemicon) in PB + 0.1% BSA-C at 4 °C. After washing in PB + 0.1% BSA-C, the sections were incubated for 4 h at room temperature in biotinylated secondary antibody (goat α-rabbit, Jackson Laboratories). To reveal this labeling, slices were incubated for 2 h in avidin biotin peroxidase complex (ABC Elite, Vector Laboratories), followed by 3,3′-diaminobenzidine tetrachloride (Vector Laboratories Kit) for 10 to 20 min. The sections were then postfixed overnight in 2.5% glutaraldehyde, washed in 0.1 M PB, postfixed in osmium tetroxide for 1 h, dehydrated, and embedded in epoxy resin.

Serial sections were cut at 40-nm thickness and collected on single-slot grids, then contrasted by incubating for 35 min in 5% uranyl acetate solution, followed by 25 min in Reynolds solution. Serial images of the labeled structures were then collected with a digital camera (MegaView III, SIS) mounted on a JEOL 100 CXII transmission electron microscope, at a 19.000× magnification, at a filament voltage of 80 kV.

### Statistical analysis.

All statistical analyses were performed using Statview 5.0.1. for Mac. For all comparisons, ANOVA was performed followed by the Fisher post hoc test, when appropriate. Differences were considered statistically significant at *p* < 0.05.

## Supporting Information

Figure S1 Cdk5 Is Expressed in NPCs upon Neuronal Differentiation and in the Adult Hippocampus (A) RT-PCR of *cdk5* and its coactivator *p35* using RNA derived from rat adult NPCs showed up-regulation of *cdk5* 1 d upon the induction of neuronal differentiation using retinoic acid (RA) and forskolin (FSK). The expression pattern of cdk5 and p35 protein was analyzed using western blots (lower panel) 1, 3, 5, and 7 d after induction of neuronal differentiation with RA and FSK.
(B) The 35S-labeled antisense riboprobes against *cdk5* detected mRNA expression (red and gray in inset) throughout the hippocampus (nuclei counterstained with bisbenzimide in blue). CA1–3, cornu ammonis area 1 and 3; DG, dentate gyrus; P, proliferation conditions with FGF-2.
(5.31 MB TIF)

Figure S2 shRNA-Mediated Knockdown of Cdk5 Recapitulates the *DNcdk5*-Phenotype (A and B) 293T cells were cotransformed with a cdk5-GFP fusion protein and a vector expressing a control shRNA (left panel in [A]) or shRNA against *cdk5* (2–1sir, left panel in [A]). Note the reduction in GFP signal in (B). Western blot analyses showed a clear reduction in cdk5 protein after transformation with 2–1sir (lane 3) compared to endogenous cdk5 control levels (lane 2). The specificity of the cdk5 band was tested with overexpression of cdk5 (lane 1).
(C) Control cells with an empty U6-cassette (left panel) expressing GFP from the CAG-promoter showed regular granule cell morphology 4 wk after virus injection. In clear contrast, cells expressing retrovirus-mediated 2–1sir (right panel) showed aberrant dendritic orientation 4 wk after retroviral injection, similar to the neuronal phenotype after *DNcdk5* overexpression. Nuclei were counterstained with DAPI (blue). Scale bar represents 20 μm. HL, hilus.
(2.73 MB TIF)

Figure S3 Spines on Aberrant Cdk5-Deficient Dendrites Are in Close Apposition to the Presynaptic Protein Synapsin Confocal images of cells expressing GFP only (A), *cdk5* (B), or *DNcdk5* with aberrant dendrites extending into the hilus (C). Arrows point toward synapsin-labeled punctae in close proximity to GFP-labeled spines. Note the GFP-labeled cell body in the GCL in (C) (arrowhead). Insets show a single channel for GFP signal.
(1.13 MB TIF)

## Abbreviations

BrdU: bromo-deoxy-uridine
cdk5: cyclin-dependent kinase 5
DCX: doublecortin
GCL: granule cell layer
GFP: green fluorescent protein
ML: molecular layer
NPC: neural stem and progenitor cell
RFP: red fluorescent protein
shRNA: small hairpin RNA
